# Methicillin- and Vancomycin-Resistant *Staphylococcus aureus* (MRSA and VRSA) in Raw and Cooked Buffalo Meat Products

**DOI:** 10.3390/foods15132254

**Published:** 2026-06-23

**Authors:** Mennat-Allah Ahmed Diaa, Amira Ibrahim Zakaria, Hazem Ramadan, Kálmán Imre, Adriana Morar, Khalid Ibrahim Sallam

**Affiliations:** 1 Department of Food Hygiene, Safety, and Technology, Faculty of Veterinary Medicine, Mansoura University, Mansoura 35516, Egypt; mennadiaa2022@yahoo.com (M.-A.A.D.); amirazakaria@mans.edu.eg (A.I.Z.); 2 Hygiene and Zoonoses Department, Faculty of Veterinary Medicine, Mansoura University, Mansoura 35516, Egypt; hazem_hassan@mans.edu.eg; 3 Faculty of Veterinary Medicine, University of Life Sciences “King Mihai I” from Timisoara, 300645 Timisoara, Romania; adrianamorar@usvt.ro

**Keywords:** *Staphylococcus aureus*, MRSA, VRSA, MDR, buffalo meat, kofta sandwiches, Fried liver, PCR, enterotoxins

## Abstract

Buffalo meat is widely consumed in Egypt; however, it may pose serious food safety risks due to microbial contamination during handling, preparation, and processing. This study investigated the prevalence and characterization of multidrug-resistant (MDR) enterotoxigenic *Staphylococcus aureus* in raw ground buffalo meat and ready-to-eat (RTE) kofta and liver sandwiches marketed in Mansoura, Egypt. *S. aureus* was detected in 62% (62/100) of raw buffalo ground meat, 41% (41/100) of RTE kofta, and 60% (60/100) of RTE liver samples, with an overall prevalence of 54.3% (163/300). All 660 isolates were confirmed as *S. aureus* via *nuc* gene detection, among which 46.8% (309/660) were *mecA*-positive and verified as methicillin-resistant (MRSA), and 21.8% (144/660) were *vanA*-positive and verified as vancomycin-resistant (VRSA). Enterotoxigenic strains were identified in 42.7% (282/660) of isolates, with the *sea* gene being most prevalent (67.7%; 191/282), followed by *seb* (58.2%; 164/282) and *sec* (39.7%; 112/282). The highest frequency of enterotoxigenic strains occurred in raw ground meat (47.2%), followed by kofta (45.1%) and liver (36%). Antimicrobial susceptibility testing against 15 antibiotics revealed that 7.6% (50/660) of isolates were extensively drug-resistant (XDR) with a MAR index of 0.9, while 82.9% (547/660) were MDR with MAR values between 0.3 and 0.7, indicating exposure to environments of intensive antibiotic use. The present findings highlight a high contamination level of buffalo meat products with MDR enterotoxigenic MRSA and VRSA, representing a significant public health hazard. Implementation of strict hygiene measures, wise antibiotic usage, and continuous surveillance is essential to control their dissemination through the food chain.

## 1. Introduction

The water buffalo (*Bubalus bubalis*) is a significant source of red meat in Egypt and many other nations, including India, Pakistan, Bulgaria, Italy, and Australia. Buffaloes account for between 45 and 50 percent of Egypt’s entire red meat supply, making them a significant contributor to the country’s meat industry [1]. Buffalo meat is different from that of beef. Buffalo meat has a darker color and less intramuscular fat, which resembles milky white fat, than beef’s yellow-white fat. Additionally, it has a lower-cholesterol alternative to beef, along with less collagen and a lower pH, which increases its tenderness [2].

Buffalo meat constitutes the second-largest component of red meat consumption in Egypt after cattle meat. Between 2008 and 2022, cattle and buffalo meat accounted for average shares of 48.6% and 37.9% of total red meat consumption, respectively, highlighting the substantial contribution of buffalo production to meeting the country’s demand for animal-derived protein. Buffalo meat and edible offal, particularly liver, are widely consumed by Egyptians, especially in rural and semi-urban communities. In recent years, however, rapid urbanization and the increasing participation of women in the workforce have altered dietary habits, leading to a growing demand for convenient ready-to-eat (RTE) food products [3]. Among these, buffalo meat sandwiches have gained considerable popularity due to their affordability, palatability, and convenience for consumers seeking meals away from home. Nevertheless, RTE meat products may represent a significant public health concern because they are susceptible to microbial contamination during slaughtering, handling, preparation, processing, and retail distribution. Given the widespread consumption of buffalo-derived products and their potential role in the transmission of foodborne pathogens, these products were selected for investigation in the present study.

Ground buffalo meat, buffalo kofta sandwiches, and buffalo liver sandwiches were selected because they are among the most commonly consumed meat products in Egypt. These products represent both raw and ready-to-eat meat categories and are susceptible to contamination at different stages of processing and handling. Therefore, they provide a suitable model for evaluating the occurrence and public health significance of *Staphylococcus aureus* in the food chain.

*Staphylococcus aureus* (*S. aureus*) is one of the most prevalent foodborne pathogens, particularly in fresh and ready-to-eat (RTE) products. It is considered the third most significant cause of foodborne illnesses globally. This Gram-positive bacterium acts as both a commensal organism and an opportunistic pathogen, colonizing up to 80% of healthy individuals. A combination of toxin production, virulence factors, invasiveness, and resistance to multiple antibiotics drives its pathogenicity [4,5]. Workers in food establishments and restaurants are the main source of *Staphylococcus aureus* since it is a prevalent commensal organism found on the skin and mucosal membranes of the nose and throat, with estimates ranging from 20 to 30% for persistent colonization and 60% for intermittent colonization [4,5].

Staphylococcal foodborne intoxication (SFI) is one of the most concerning foodborne outbreaks due to its ability to produce a wide variety of heat-stable enterotoxins (SEs) [6,7]. Toxin levels required to cause SFI symptoms range from 20 ng to 1000 ng, which is equivalent to at least 10^5^ CFU/g of food [8]. Approximately 95% of SFI epidemics are caused by the traditional *sea*, *seb*, *sec*, *sed*, and *see*. The primary signs of SFI include nausea, vomiting, and abdominal cramps; nonetheless, diarrhea and varying levels of dehydration can also occur [9].

Antimicrobial resistance (AMR) is one of the biggest problems that results from the misuse and overuse of antibiotics. According to the FAO/WHO [10], at least 25% of the foodborne isolates exhibit resistance to at least one class of antibiotics. Bacteria are classified according to the degree of resistance as follows: pan-drug resistant (PDR), extensively drug-resistant (XDR), and multidrug-resistant (MDR) [11].

The most critical concern is the emergence of methicillin-resistant *Staphylococcus aureus* (MRSA), which was historically confined to the hospital setting between 1930 and 1980. However, by the late 1990s, MRSA infections began appearing among healthy individuals in the community, raising public health concerns due to limited treatment options and increased morbidity [12]. Methicillin-resistant *S. aureus* (MRSA) strains have been detected in a variety of food sources. It is distinguished by its resistance to β-lactam antibiotics, such as cephalosporins and penicillin. The acquisition of the *mecA* gene is the main cause of the resistance mechanism [13].

In recent decades, vancomycin and linezolid have remained the antibiotics of last resort for the treatment of MRSA infections; nevertheless, vancomycin-resistant *S. aureus* (VRSA) strains have emerged and spread worldwide, posing a serious threat to public health due to the limited therapeutic options available for their treatment [14,15,16]. Vancomycin resistance is associated with several van gene clusters, including *vanA*, *vanB*, *vanD*, *vanF*, *vanI*, and *vanM* [14]. Given the widespread consumption of buffalo-derived products in Egypt and the potential role of raw and ready-to-eat meat products as reservoirs and transmission vehicles of antimicrobial-resistant *S. aureus*, the present study aimed to determine the prevalence, antimicrobial resistance patterns, and molecular characteristics of *S. aureus* isolates recovered from raw ground buffalo meat, ready-to-eat kofta sandwiches, and buffalo liver sandwiches, with particular emphasis on the detection and characterization of methicillin-resistant and vancomycin-resistant strains (MRSA and VRSA).

## 2. Materials and Methods

### 2.1. Collection of Samples

Three hundred samples comprising 100 raw ground buffalo meat and 200 RTE meat products (100 each of liver sandwiches and kofta sandwiches) were purchased from 10 butcher shops, 8 fast-food restaurants, and 12 RTE food outlets (cafeterias and street vendors) with different hygienic standards distributed in 6 neighborhoods in Mansoura city, Egypt, from October 2024 to July 2025. All products originated from buffalo carcasses. Each sample was put separately in a sterile plastic bag, marked, tightly sealed, placed in an ice box, and transferred immediately to the laboratory of the Food Hygiene and Control Department, Faculty of Veterinary Medicine, Mansoura University, Egypt. Samples were then subjected to bacteriological examination for *S. aureus* isolation and characterization.

### 2.2. Isolation, Identification, and Enumeration of S. aureus

*Staphylococcus aureus* was isolated using the International Organization for Standardization’s guidelines “ISO” 6888-1:2021 [17]. Briefly, 25 g were aseptically taken from raw ground meat and sandwich core (meat/liver, sauces, and vegetables) using a sterile scissor and homogenized in 225 mL of 0.1% tryptone soy broth (Oxoid, CM0129; Oxoid Ltd., Basingstoke, Hampshire, UK) for 60 s in a laboratory blender to create the initial dilution. Ten-fold serial dilutions were prepared from the initial dilution. The chosen dilution was then distributed and cultivated on duplicate plates of Baird-Parker selective agar (Oxoid CM275; Oxoid Ltd., Basingstoke, Hampshire, UK) enriched with egg-yolk tellurite emulsion using the spread method. Following that, the plates were incubated at 37 °C for 48 h. It was assumed that isolates exhibiting perfect colonies—shiny, black, round, smooth, convex, and 2–3 mm in diameter with an opaque halo encircled by a 25 mm clearing zone—were *S. aureus*. For each presumptive positive sample, three to five colonies exhibiting the typical morphological characteristics of *Staphylococcus aureus* on Baird-Parker agar were selected from the countable plates and subjected to biochemical and molecular confirmation. Identification by biochemical tests includes coagulase production, catalase activity, detection of hemolysis, mannitol fermentation test, and deoxyribonuclease (DNase). Only coagulase-positive *S. aureus* colonies were considered and were further subjected to polymerase chain reaction (PCR) to confirm the positive *S. aureus* isolate through the detection of the *nuc* gene. The *nuc* gene in *S. aureus* encodes a heat-stable nuclease (thermonuclease), which is commonly used as a molecular target gene for the rapid detection of *S. aureus*.

### 2.3. Genomic DNA Extraction

Genomic DNA of the coagulase-positive *S. aureus* isolates recovered from the tested food samples, as well as from the control positive (*S. aureus* ATCC 43300) and control negative (*E. coli* K-12 DH5α). was extracted using the GeneJET Genomic DNA Purification Kit (K0721, Thermo Fisher Scientific Baltics UAB, Vilnius, Lithuania) according to the manufacturer’s instructions. Briefly, a fresh bacterial culture was harvested and subjected to cell lysis using the provided lysis solution and Proteinase K. Following lysis, DNA was purified through silica membrane spin columns, washed to remove contaminants, and subsequently eluted in the supplied elution buffer. The concentration and purity of the extracted DNA were assessed spectrophotometrically, and the purified DNA was stored at −20 °C until further molecular analysis.

### 2.4. Molecular Characterization of S. aureus

#### 2.4.1. Detection of *nuc* Gene

All isolated presumed *S. aureus* colonies were verified through the detection of the staphylococcal thermostable nuclease marker gene (*nuc* gene) using a PCR technique. The particular primer set specific to the *nuc* gene is shown in Table 1. The *nuc* gene is amplified using the previously reported PCR cycling conditions [18]. A SimpliAmp™ Thermal Cycler (Thermo Fisher Scientific Inc.) was employed for gene amplification in a 25-μL reaction mixture containing 2.0 μL of *S. aureus* genomic DNA template, 1.0 μL of each of the forward and reverse primers, 2.5 μL of 2 mM dNTPs, 2.5 μL of PCR buffer (10×), 0.5 μL of Taq polymerase, 2 μL of 25 mM MgCl_2_, and 13.5 μL of RNase/DNase-free water (Thermo Fisher Scientific Baltics UAB, Vilnius, Lithuania). The PCR amplification settings were as follows: 5 min of initial DNA denaturation at 95 °C; 35 cycles of denaturation at 95 °C for 30 s, annealing at 57 °C for 40 s, and extension at 72 °C for 90 s; and a final extension at 72 °C for 5 min. After amplification, 8 μL of the amplified PCR product was loaded in a 1.5% agarose gel containing 0.5 μg/mL ethidium bromide (Sigma-Aldrich, Co., St. Louis, MO, USA) and electrophoresed by Cleaver Scientific gel electrophoresis system (Cleaver Scientific Ltd., Rugby, Warwickshire, UK) using 1× TBE running buffer (Thermo Fisher Scientific, Vilnius, Lithuania) for 50 min at a voltage of 100 V. Under ultraviolet light, the amplified DNA bands were separated and seen and visualized at the anticipated molecular size of 279 bp.

#### 2.4.2. Molecular Identification of MRSA and VRSA

The antimicrobial resistance genes comprising methicillin-resistant (*mecA*) and vancomycin-resistant (*vanA*) genes were detected in *S. aureus* isolates by multiplex PCR using the previously constructed primer sets (Table 1) that were generated to yield a 1200 bp *mecA gene* [19], and a 235 bp *vanA* gene [20]. PCR mixture of 50-μL containing 4.0 μL of *S. aureus* DNA template, 1.0 μL of each of the forward and reverse primer, 5 μL of 2 mM dNTPs, 5 μL of PCR buffer (10×), 1.0 μL of Taq polymerase, 4 μL of 25 mM MgCl_2_, and 27 μL of RNase/DNase-free water (Thermo Fisher Scientific Baltics UAB, Vilnius, Lithuania) was prepared for the target genes amplification using SimpliAmp™ Thermal Cycler (Thermo Fisher Scientific Inc.). The cycling conditions involved an initial denaturation at 95 °C for 5 min, 30 cycles of denaturation at 95 °C for 45 s, annealing at 56 °C for 45 s, extension at 72 °C for 80 s (for *mecA* gene) and 30 s (for *vanA* gene) followed by final extension at 72 °C for 10 min (for *mecA*) and 5 min (for *vanA*). Ten microliters of the amplified DNA was loaded in a 1.5% agarose gel containing 0.5 μg/mL ethidium bromide (Sigma-Aldrich, Co., St. Louis, MO, USA) and electrophoresed by Cleaver Scientific gel electrophoresis (Cleaver Scientific LTD) using 1× TBE running buffer for 50 min at a voltage of 100 V.

#### 2.4.3. Detection of Enterotoxin Virulence Genes

The molecularly verified *S. aureus* strains, identified by the *nuc* gene, were further screened using multiplex PCR targeting the *S. aureus* enterotoxin genes *sea*, *seb*, and *sec*. Primer sequences of tested *S. aureus* enterotoxin genes that are expected to generate molecular sizes of 500 bp, 600 bp, and 300 bp for *sea*, *seb*, and *sec* genes, respectively, are mentioned in Table 1.

**Table 1:** Sequences of primer sets used for detecting marker, virulence, and antimicrobial resistance genes among coagulase-positive *S. aureus* strains isolated.

Target Genes	Oligonucleotide Sequence (5′ → 3′)	Product Size (bp)	References
***nuc***	F: 5′-GCGATTGATGGTGATACGGTT-3′ R: -5′AGCCAAGCCTTGACGAACTAAAG-3′	279	Wang et al. [21]
***Sea***	F: 5′-TGCAGGGAACAGCTTTAGGCAA-3′ R: 5′-GATTAATCCCCTCTGAACCTTCC-3′	500	Sallam et al. [19]
***seb***	F: 5′-CCTAAACCAGATGAGTTGCACAAAGCG-3′ R: 5′-TCCTGGTGCAGGCATCATGTCATA-3′	600	
***sec***	F: 5′-GCCAGATGAGTTGCACAAATC-3′ R: 5-′CCACCTGTAACTTTACCTAC-3′	300	
***mecA***	F: 5′-GATTGGGATCATAGCGTCA-3′ R: 5′-CAGTATTTCACCTTGTCCG-3′	1200	
***vanA***	F: 5′-GGGAAAACGACAATTGC-3′ R: 5′-GTACAATGCGGCCGTTA-3′	235	Elsalkh et al. [20]

In a thermal cycler, DNA amplification was carried out under the following cycling conditions: 5 min of initial denaturation at 95 °C, 35 cycles of denaturation at 95 °C for 40 s, 40 s of annealing at 57 °C, 45 s of extension at 72 °C, and 7 min of final extension at 72 °C. PCR product aliquots were placed onto a 1.5% agarose gel, electrophoresed for 50 min at 100 V, visualized, and photographed under ultraviolet light.

### 2.5. Antimicrobial Resistance Profile of S. aureus Isolates Recovered from Ground Meat and RTE Kofta and Liver Sandwiches

The antimicrobial resistance profile of all molecularly verified *S. aureus* isolates (*n* = 660) recovered from 163 positive samples was carried out by testing against 15 antimicrobial agents constituting 12 antimicrobial classes according to The Clinical and Laboratory Standards Institute’s (CLSI) guidelines [22]. Vancomycin and oxacillin were tested using the agar dilution method to determine minimum inhibitory concentrations (MICs), as MIC-based testing provides a more reliable assessment of susceptibility to these critical antimicrobials and facilitates accurate identification of VRSA and MRSA strains. The remaining antimicrobial agents were evaluated using the agar disk diffusion method using Mueller-Hinton agar (MH; CM0337; Oxoid Ltd., Basingstoke, UK) and Antimicrobial Susceptibility Discs (Oxoid Ltd., Basingstoke, UK). Vancomycin MIC breakpoints of ≤2, 4–8, and ≥16 μg/mL for *S. aureus* strains were confirmed to be susceptible, intermediate, and resistant, respectively [22]. Oxacillin-sensitive *S. aureus* were defined as having MIC breakpoints of ≤2 µg/mL, while oxacillin-resistant *S. aureus* were defined as having breakpoints of ≥4 µg/mL. The following antimicrobials tested were sulfamethoxazole-trimethoprim (25 µg), vancomycin, rifampin (30 µg), kanamycin (30 µg), azithromycin (30 µg), ampicillin (25 µg), gentamicin (30 µg), oxacillin, tetracycline (30 µg), clindamycin (10 µg), cefotaxime (30 µg), nitrofurantoin (300 µg), levofloxacin (5 µg), ciprofloxacin (10 µg), and linezolid (30 µg). After 24 h of incubation at 35 °C, the MIC breakpoints (μg/mL) and inhibition zone diameter breakpoints (mm) for each antibiotic were established according to the CLSI [22] guidelines. According to their AMR profiles, the tested *S. aureus* isolates were divided into three categories: pan-drug-resistant (PDR), which demonstrated complete resistance to all antimicrobials in all antimicrobial classes examined; extensively drug-resistant (XDR), which exhibited resistance to most available antibiotics, specifically non-susceptible to at least one agent in all but two or fewer antimicrobial categories (i.e., the isolates is susceptible to one or two antimicrobial classes); and multidrug-resistant (MDR), which was resistant to at least three antimicrobials [11]. The MAR index for each *S. aureus* isolate was calculated by dividing the total number of antimicrobials tested by the number of antimicrobial agents to which the isolate exhibited resistance. A high-risk source of contamination, where antimicrobial drugs are regularly used, is indicated by a MAR index greater than 0.2 [23].

### 2.6. Statistical Analysis

Statistical analyses were performed using IBM SPSS Statistics for Windows, Version 26.0 (IBM Corp., Armonk, NY, USA). *S. aureus* counts were expressed as mean ± standard error (SE). Differences in mean bacterial counts among raw ground buffalo meat, ready-to-eat (RTE) kofta sandwiches, and RTE liver sandwiches were evaluated using one-way analysis of variance (ANOVA) followed by Tukey’s post hoc test for multiple comparisons. Associations between sample source (raw versus RTE products) and the prevalence of antimicrobial resistance genes (*mecA* and *vanA*) and enterotoxin genes (*sea*, *seb*, and *sec*) were assessed using the Chi-square (χ^2^) test of independence. When expected cell frequencies were <5, Fisher’s exact test was applied. Differences were considered statistically significant at *p* < 0.05. 

## 3. Results and Discussion

### 3.1. Prevalence and Counts of Staphylococcus aureus in Raw and Cooked Buffalo Meat Products

**Figure 1:**
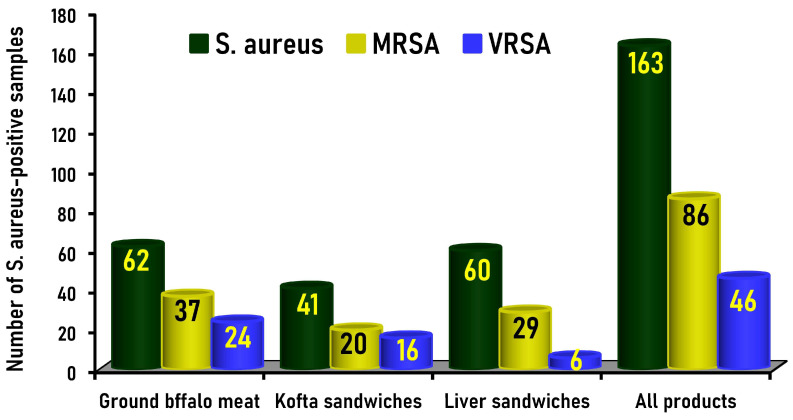
Prevalence and distribution of nuclease (*nuc*)-positive *S. aureus* isolates, methicillin-resistant *S. aureus* (MRSA), and vancomycin-resistant *S. aureus* (VRSA) in the examined samples of raw ground buffalo meat, grilled kofta sandwiches, and RTE fried liver sandwiches, showing the number of meat samples positive for *S. aureus*, MRSA, and VRSA.

Meat products with poor hygienic measures can lead to an increase in the rate of contamination with *S. aureus*. In the present study, 62% (62/100), 41% (41/100), and 60% (60/100) of raw ground beef, RTE kofta sandwiches, and RTE liver sandwiches, respectively, were contaminated with *S. aureus*, with an overall prevalence rate of 54.3% (163/300) (Figure 1).

According to the Commission Regulation (EC) No 2073/2005 of the EU, *S. aureus* counts of >10^3^ CFU/g in ready-to-eat foods are considered unsatisfactory, while a count of more than 10^5^ CFU/g in foods can produce enough enterotoxin to cause a hazardous risk of food poisoning. The mean *S. aureus* counts in raw ground beef, RTE kofta sandwiches, and RTE fried liver samples were 7.94 × 10^4^, 2.51 × 10^4^, and 6.31 × 10^3^ CFU/g, respectively. Interestingly, 53, 38, and 45% of raw ground beef, RTE kofta sandwiches, and RTE liver samples (Table 2) surpassed the maximum permissible limit of 10^3^ CFU/g set by Egypt’s National Food Safety Authority and were deemed unfit for human consumption.

Raw ground beef samples showed the highest mean count (7.94 × 10^4^ CFU/g), with more than half (53%) of the samples tested being unacceptable since they exceeded the maximal acceptable limit. This indicates that raw ground beef is highly liable to bacterial contamination, most likely due to extensive exposure during handling, grinding, and processing. Although kofta sandwiches exhibited a mean lower *S. aureus* count (2.51 × 10^4^) compared to raw ground beef, they still represent a significant public health concern. This is particularly relevant in Egypt, where kofta sandwiches are widely consumed and frequently prepared, thus increasing the potential risk of exposure to foodborne pathogens if proper hygienic measures are not strictly followed. On the other hand, liver sandwiches revealed a higher contamination rate than kofta sandwiches, which may be attributed to the natural load of liver tissue combined with inadequate cooking or cross-contamination during preparation. In general, the contamination and unacceptable rates followed the order: raw ground beef > liver sandwiches > kofta sandwiches, which seems to be reasonable, as it is not subjected to heat treatment.

Statistical analysis revealed significant differences (*p* < 0.01) in mean *S. aureus* counts among the examined buffalo-derived products. Raw ground buffalo meat exhibited significantly higher counts than both RTE kofta and RTE liver sandwiches, reflecting the effect of heat treatment and handling practices on microbial load reduction. However, the persistence of substantial contamination levels in RTE products indicates possible post-processing contamination and inadequate hygienic practices during preparation and serving.

The occurrence of *S. aureus* observed in the present study is consistent with that obtained from the recent study conducted in our laboratory, which indicated that 56% (56/100) of ready-to-eat meat pizza distributed in Mansoura City were contaminated with *S. aureus* [20]. Likewise, a prevalence rate of 54.45% (55/101) was reported for *S. aureus*, with a mean bacterial count of 3.40 ± 0.63 log_10_ cfu/g, in raw beef samples tested in Ethiopia [24]. Conversely, lower prevalence rates were reported in various meat products worldwide. In China, Wu et al. [25] indicated that 50.4% (63/125) of raw beef and 25.0% (3/12) of RTE beef samples were positive for *S. aureus*. In Turkey, 30.5% (61/200) of examined raw beef samples were positive for *S. aureus* [18], while in Bangladesh, Islam et al. [26] revealed that 17.1% (6 of 35) of raw meat and meat products examined were positive for *S. aureus*, while 26 of 112 (23.2%) RTE street-vended foods were contaminated with *S. aureus*. A much lower prevalence rate of 6.9% (2/29) was demonstrated for beef samples from retail stores in Iowa in the United States [27], although an extremely lower prevalence rate of 1.14 (39/3417) of RTE meat products was demonstrated in China by Yang et al. [28]. On the contrary, higher than our findings, Mahros et al. [29] indicated that 87.5% and 78.1% of the examined beef burger and hot dog sandwiches were contaminated with *S. aureus*, with mean counts of 3.6 × 10^3^ and 4.4 × 10^3^ CFU/g, respectively. A higher prevalence rate of 69.2% (18/26) was also detected in ground beef from Georgia, USA [30]. The inconsistency in the prevalence and counts of *S. aureus* among RTE meat products may be explained by differences in raw material quality, hygienic practices during processing and vending, type of product and degree of handling, post-cooking cross-contamination, environmental and storage conditions, and methodological variations in sampling and detection.

The variation in contamination rates among the examined products is likely influenced by multiple factors. The comparatively lower prevalence observed in ready-to-eat products may be partly attributed to the bacterial reduction achieved during cooking. Nevertheless, the recovery of *S. aureus* from ready-to-eat sandwiches indicates that contamination can still occur after cooking through improper handling, contaminated utensils, food-contact surfaces, or inadequate hygienic practices during preparation and serving. In contrast, the higher contamination rate detected in ground buffalo meat may reflect contamination introduced at various stages of the food chain, including slaughtering, processing, grinding, transportation, and retail display. Therefore, the observed differences are likely the result of both production-chain contamination and post-processing hygienic deficiencies rather than a single contributing factor.

Interpretation of prevalence data across studies should be approached with caution because direct comparisons may be affected by numerous factors beyond the actual occurrence of *Staphylococcus aureus*. These factors include differences in meat product categories (raw versus ready-to-eat products), sampling strategies, sample sizes, study periods, laboratory methodologies, production systems, hygienic practices, antimicrobial usage patterns, and national food safety regulations. Furthermore, variations in livestock management and regulatory oversight among countries may substantially influence contamination levels. Therefore, the differences observed between the present study and previous reports likely reflect a combination of these factors rather than a single underlying cause.

### 3.2. Genetic Characterization of S. aureus Isolates Retrieved from Examined Raw and Cooked Meat Samples

#### 3.2.1. Detection and Characterization of Methicillin- and Vancomycin-Resistant *Staphylococcus aureus* (MRSA and VRSA) Among *S. aureus* Isolates

**Figure 2:**
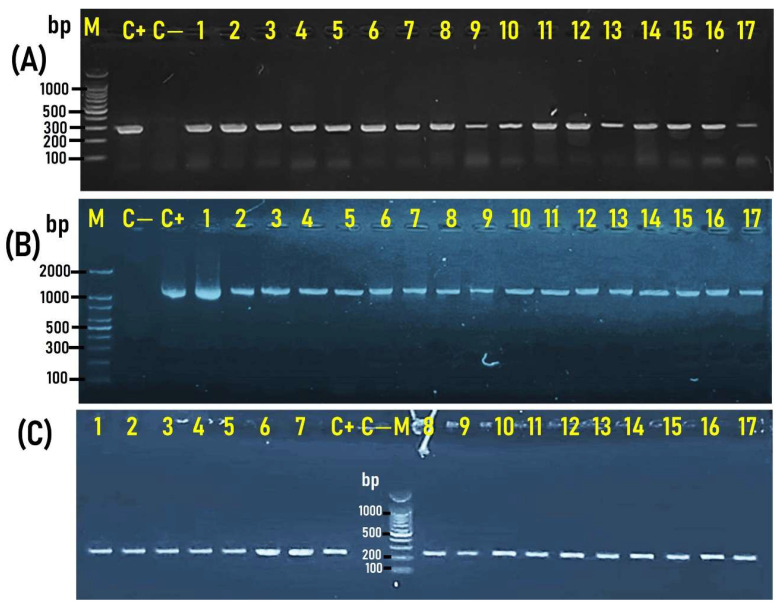
Representative agarose gel electrophoresis for PCR demonstrated amplified bands of the *nuc*, *mecA*, and *vanA* genes. (**A**): Amplified bands of the *nuc* gene-positive *S. aureus* (Lanes 1–17), detected at the molecular size of 279 bp. (**B**): The *mecA* gene, which is the specific marker for methicillin-resistant *S. aureus* (MRSA) (Lanes 1 to 17), is detected at the molecular size of 1200 bp. (**C**): The *vanA* gene, the specific marker for vancomycin-resistant *S. aureus* (VRSA), is detected at the molecular size of 235 bp. M: DNA marker (100 bp gene ladder). C+: Control positive (*S. aureus* ATCC 43300), C−: Control negative (*E. coli* K-12 DH5α). Eight microliters of the PCR product were separated by electrophoresis on a 1.5% agarose gel and visualized under UV light.

Six hundred sixty *S. aureus* isolates recovered from 163 *S. aureus*-positive food samples, comprising 248 isolates from raw ground beef, 184 from RTE kofta sandwiches, and 228 from RTE fried liver sandwiches, were molecularly confirmed by amplification of the *nuc* gene, which was distinguished at the anticipated size of 278 bp DNA (Figure 2A). The *nuc* gene encodes the thermonuclease enzyme and has been widely used as a target marker to identify *S. aureus* [17].

**Figure 3:**
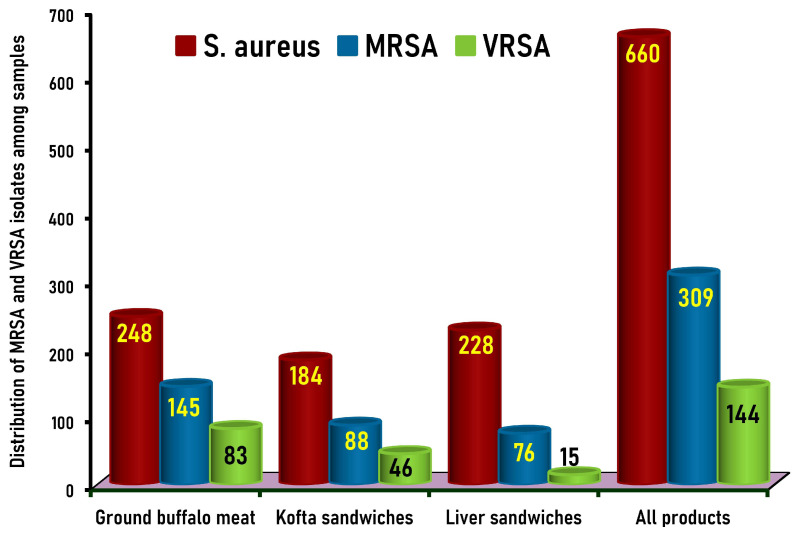
Distribution of the identified *S. aureus*, MRSA, and VRSA isolates among the tested meat product samples.

For the confirmation of MRSA, all *S. aureus* isolates (*n* = 660) were subjected to PCR to detect the presence of the mecA gene, which is the gold-standard marker for methicillin resistance. The mecA gene was successfully amplified at the expected molecular size of 1200 bp (Figure 2B) in 309 of the 660 isolates tested, indicating an overall MRSA prevalence rate of 46.8% among the isolates (Figure 3). Such a high rate was distributed differently across the different meat products. Interestingly, 58.8% (145/248) of *S. aureus* isolates recovered from raw ground beef tested in the present study were mecA-positive (Figure 3), which is consistent with findings reported in a survey carried out in China by Wu et al. [25], where 54.8% (34/62) of *S. aureus* isolates from raw meat were confirmed as methicillin-resistant *S. aureus* (MRSA). On the other hand, 47.8% (88/184) and 33.3% (76/228) of the isolates recovered from examined RTE kofta sandwiches and RTE liver sandwiches, respectively, were *mecA*-positive and verified as MRSA (Figure 2B).

Chi-square analysis demonstrated a significant association (*p* < 0.05) between sample source and the prevalence of *mecA*- and *vanA*-positive isolates. Raw ground buffalo meat showed significantly higher frequencies of MRSA and VRSA compared with RTE products, suggesting that raw meat may serve as a more important reservoir of antimicrobial-resistant *S. aureus* strains. Nevertheless, the detection of MRSA and VRSA in RTE products remains a considerable public health concern because these products are consumed without further heat treatment.

The current result is broadly comparable to those reported in a recent study from our laboratory, which revealed that 45% (72/160) of *S. aureus* isolates recovered from RTE meat pizza were confirmed as MRSA [20], while Mahros et al. [29] reported lower prevalence rates of 25% (35/140) and 16% (8/50) of coagulase-positive *S. aureus* isolates recovered from beef burger and hot dog sandwiches, respectively, were positive for the *mecA* gene and confirmed as MRSA, with an overall rate of 22.6% (43/190) among the isolates. Likewise, Islam et al. [26] reported that 8 (30.8%) of 26 *S. aureus* isolates recovered from RTE food were verified as MRSA. A much lower MRSA prevalence rate of 12.9% (8/62) was found among isolates from RTE meat tested in China [25].

The contribution of MRSA-positive isolates at the sample level in the present study demonstrated that 86 out of 300 (28.7%) meat product samples were positive for MRSA, which comprised 37 (37%) of the raw ground beef, 20 (20%) of the RTE kofta sandwiches, and 29 (29%) of the RTE grilled liver sandwiches (Figure 1). These findings underscore the significant prevalence of MRSA in the food supply, with RTE beef products posing a serious public health concern. The prevalence of MRSA in the examined samples in this study is comparable to that reported by Elsalkh et al. [20], who detected MRSA at 35% (35/100) of examined RTE meat pizza samples. Although in the USA, Wells and Juett [31] identified MRSA in 27.8% (5/18) of raw meat samples collected from various sites in Kentucky.

Lower prevalence rates of MRSA among raw and RTE beef were globally described in numerous studies. For instance, in Iran, Safarpoor Dehkordi et al. [32] recorded that 6 (15.8%) of 38 raw meat samples and 5 (16.1%) of 31 of RTE meat barbecue samples, respectively, were confirmed as MRSA-positive. In the Netherlands, de Boer et al. [33] isolated MRSA from 10.6% (42/395) of beef samples, while in Bangladesh, Islam et al. [26] reported that only 1 (2.9%) of 35 raw beef samples was MRSA-positive, although they found that 7 (6.3%) of 112 RTE street-vended foods harbored MRSA. In the USA, Ge et al. [34] demonstrated that MRSA was present in 1.9% of 66/3520 retail meat samples. In Korea, 0.3% (4/1587) of domestic and imported beef meat [35], and 1.0% (9/890) of raw beef meat samples [36] were positive for MRSA.

Vancomycin is regarded as a last-line antibiotic for the treatment of serious *S. aureus* infections, and the detection of resistance to this critical drug in the food supply chain highlights the need for continuous surveillance and stringent food safety measures. The presence of the *vanA* gene, a key determinant of high-level vancomycin resistance, was investigated using PCR to confirm the identity of vancomycin-resistant *Staphylococcus aureus* (VRSA) isolates. In the current study, the amplification of the *vanA* gene was successfully detected in positive samples, yielding a PCR product at the anticipated molecular size of 235 bp (Figure 2C).

The molecular analysis revealed an overall VRSA prevalence rate of 21.8% (144/660) among the *S. aureus* isolates (Figure 3). This resistance was not uniformly distributed across the isolates recovered from the different meat products. Isolates from raw ground beef displayed the highest VRSA prevalence rate at 33.5% (83/248), while isolates from RTE kofta sandwiches showed a lower prevalence of 25% (46/184), and isolates from RTE liver sandwiches exhibited the lowest prevalence rate of 6.6% (15/228). Among the samples tested, VRSA exhibited relatively high prevalence rates of 24% (24/100), 16% (16/100), and 6% (6/100) in raw ground beef, RTE kofta sandwiches, and RTE liver sandwiches, with an overall contamination rate of 15.3% (46/300).

A similar prevalence rate of 15% (15/100) was recorded for VRSA in meat pizza samples, which constituted 20% (32/160) of *S. aureus* isolates recovered from RTE meat pizza [20]. However, a lower VRSA prevalence rate of 6.25% (4/64) was reported in RTE burger and shawarma sandwiches collected from Zagazig city, Egypt [37].

The variation in MRSA and VRSA prevalence in raw and ready-to-eat meat products among countries can be attributed to differences in antimicrobial use in livestock, hygiene and processing standards, and surveillance systems. Countries with extensive antibiotic use and poor hygiene often show higher contamination rates, while strict antimicrobial control and good manufacturing practices reduce prevalence. Variations in meat type, sampling methods, detection techniques, and environmental factors also contribute to discrepancies. Additionally, contamination from food handlers or processing environments may increase MRSA levels, particularly in RTE products.

The detection of multidrug-resistant *S. aureus*, including MRSA and VRSA strains, highlights the need for strict adherence to good hygienic practices during food preparation and handling, routine food safety training for food handlers, implementation of HACCP-based control systems, and strengthened antimicrobial stewardship programs in food-producing animals.

#### 3.2.2. Virulent Genes Detection in *S. aureus* Isolates Recovered from Raw and Cooked Meat Samples

*Staphylococcus aureus* enterotoxins (SEs) constitute a diverse family of low-molecular-weight, heat-stable exotoxins with potent superantigenic and emetic properties representing a significant public health hazard. These toxins encompass classical types (*sea*–*see*). Beyond food poisoning, certain SEs—especially *seb* and *sec*—have been implicated in severe illnesses like toxic shock syndrome and have been classified as potential biological warfare agents due to their extreme potency, stability, and low dose required to cause illness. Their heat and protease resistance make SEs persistent and difficult to eliminate from food matrices, posing ongoing risks to consumer health and food safety worldwide. In this context, De Buyser et al. [9] revealed that 95% of SFI cases were caused by *seb*, *sed*, *sea*, *see*, and *sec*, while Atanassova et al. [38] declared that *sea was* implicated in 80% of SFI outbreaks in the USA.

**Figure 4:**
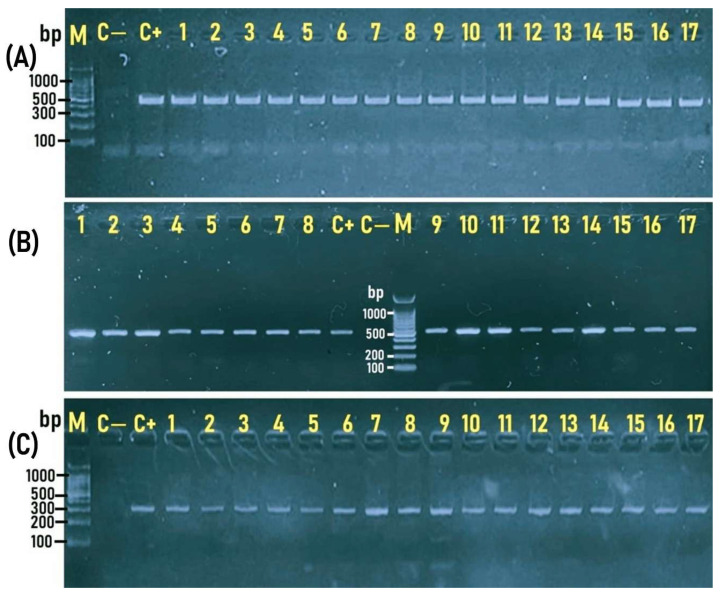
Representative agarose gel electrophoresis for multiplex PCR showing the amplified bands for *S. aureus* enterotoxin genes *sea*, *seb*, and *sec*, detected at the sizes of 500, 600, and 300 bp, respectively (**A**): Amplified bands of the *sea* gene (Lanes 1–17), detected at the molecular size of 500 bp. (**B**): The *seb* gene (Lanes 1 to 17) is detected at the molecular size of 600 bp. (**C**): The *sec* gene is detected at the expected molecular size of 235 bp. M: DNA marker (100 bp gene ladder). C+: Control positive (*S. aureus* ATCC 43300), C−: Control negative (*E. coli* K-12 DH5α). The PCR products (8 μL) were separated by electrophoresis on a 1.5% agarose gel and visualized under UV light.

The 660 *S. aureus* isolates (248 from raw ground meat, 184 from RTE kofta sandwiches, and 228 from RTE liver sandwiches) recovered in the current study were subjected to multiplex PCR for detecting various virulence factors, including SE genes such as *sea*, *seb*, and *sec*, which were identified at the expected molecular sizes of 500, 600, and 300 bp, respectively (Figure 4). The distribution of classical enterotoxin genes (*sea*, *seb*, and *sec*) among *S. aureus* isolates (*n* = 660) recovered from buffalo meat products is shown in Table 3. Among the 660 *S. aureus* isolates tested, 282 (42.7%) were positive for at least one enterotoxin gene. The highest prevalence of enterotoxigenic strains was observed in raw ground beef isolates (47.2%, 117/248), followed closely by kofta isolates (45.1%, 83/184), while liver isolates showed a comparatively lower enterotoxigenic prevalence of 36% (82/228).

Collectively, *sea* gene was determined in 67.7% (191/282) of the enterotoxigenic strains, while *seb* and *sec* were determined in 58.2% (164/282) and 39.7% (112/282) of the enterotoxigenic strains, respectively. When considering individual genes, *sea* was most common among isolates from ground beef (27/117; 23.1%) and kofta (19/83; 22.9%), while *seb* was more prevalent among liver isolates (21/82; 25.6%). The *sec* gene, however, was less frequent, detected in 10.3% (12/117) of raw ground beef isolates, 10.8% (9/83) of kofta sandwich isolates, and 13.4% (11/82) of liver sandwich isolates. The coexistence of SE genes showed that *sea*, *seb*, and *sec* were found together in 18/117 (15.4%), 15/83 (18.1%), and 16/82 (19.5%) of isolates from raw ground beef, RTE kofta sandwiches, and RTE liver sandwiches, respectively, with an overall coexistence in 49/282 (16.5%) of enterotoxigenic isolates. A dual presence of *sea* and *sec* was observed in 13/117 (11.1%), 12/83 (14.5%), and 6/82 (7.3%) of isolates from raw ground beef, RTE kofta sandwiches, and RTE liver sandwiches, respectively, with an overall rate of 31/282 (10.4%). Conversely, the coexistence of *sea* and *seb* was detected in 13/117 (11.1%), 13/83 (14.5%), and 19/82 (7.3%) of isolates obtained from raw ground beef, RTE kofta sandwiches, and RTE liver sandwiches, respectively, with an overall incidence of 61/282 (20.5%).

Statistical analysis showed a significant relationship (*p* < 0.05) between sample source and the distribution of enterotoxigenic *S. aureus* isolates. Raw ground buffalo meat harbored the highest proportion of enterotoxin-producing strains, whereas lower frequencies were observed among isolates recovered from RTE products. The occurrence of classical enterotoxin genes in both raw and RTE products highlights their potential role in staphylococcal food poisoning and emphasizes the need for improved hygienic measures throughout the food production chain.

Approximately similar findings were reported by Chen et al. [39] in China, where 64.02% (169/264) of *S. aureus* isolates recovered from meat product samples harbored at least one enterotoxigenic gene, with incidence of (11.24%), (14.79%), and (16.57%) for the classical including *sea*, *seb*, and *sec*, respectively. Similarly, the prevalence of SE genes identified among *S. aureus* isolates recovered from meat sandwiches tested was 33.6%, 17.2%, and 14.7% for *sea*, *seb*, and *sec*, respectively [29]. In contrast, a higher prevalence rate for *S. aureus* enterotoxigenic strains was reported by Wu et al. [40] in China, who detected SE genes in 108 MRSA isolates recovered from meat and meat product samples with incidence rates of 75.0% (79/108), 63.9% (68/108), and 50.9% (54/108) of *sec*, *sea*, and *seb*, respectively. Likewise, Elalkh et al. [20], in a recent study conducted in our laboratory, revealed that *sea* and *seb* were determined in 84/160 (52.5%) and 116/160 (72.5%), respectively, among *S. aureus* isolates recovered from meat pizza, although *sec* was determined at a lower rate of 6.3% (10/160). In another study conducted in Turkey, Şanlıbaba [18] revealed that 65.6% (63/96) of *S. aureus* strains recovered from raw red meat of beef, sheep, and lamb contained the SE gene, with *sea* being the highest prevalence (50.79%), followed by *sed* (25.39%) and *seb* (23.80%), although *sec* was never detected.

A limitation of the present study is that the enterotoxigenic characterization of *S. aureus* isolates was restricted to the detection of the *sea*, *seb*, and *sec* genes. Although these genes are among the most frequently implicated enterotoxins in staphylococcal food poisoning, other classical enterotoxin genes (*sed* and *see*) as well as non-classical enterotoxin and enterotoxin-like genes were not investigated. Therefore, the actual enterotoxigenic potential of the isolates may have been underestimated. Future studies employing a broader molecular screening approach are warranted to provide a more comprehensive assessment of the virulence profiles of foodborne *S. aureus* strains.

### 3.3. Antimicrobial Resistance Profile of S. aureus Isolated from Raw and Cooked Meat Products

**Figure 5:**
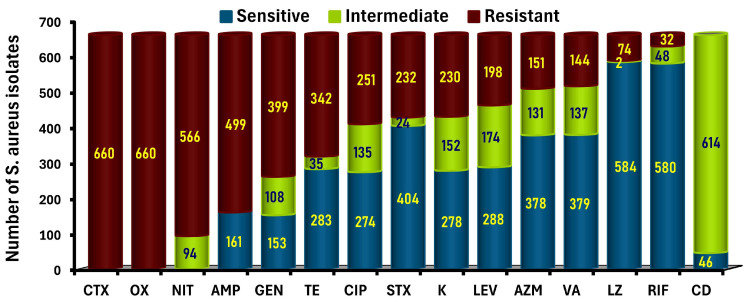
The distribution of *S. aureus* isolates according to their resistance to 15 different antibiotics from 12 classes. Each bar represents the number of isolates classified as Sensitive (blue), Intermediate (light green), or Resistant (Red) following the National Committee for Clinical Laboratory Standards (NCCLS) criteria.

*Staphylococcus aureus* isolates (*n* = 660) obtained from raw ground beef and ready-to-eat (RTE) sandwiches were tested against 15 antimicrobial agents. The results indicated that all the isolates (100%) were resistant to cefotaxime and oxacillin (Table 4 and Figure 5). High resistance rates of 85.8% (566/660), 75.6% (499/660), 60.5% (399/660), and 51.8% (342/660) were reported for nitrofurantoin, ampicillin, gentamicin, and tetracycline, respectively (Table 4 and Figure 5). Lower resistance rates of 38% (251/660), 35.2% (232/660), 34.8% (230/660), 29.5% (198/660), and 22.9% (151/660) were demonstrated for ciprofloxacin, trimethoprim/Sulfamethoxazole, kanamycin, levofloxacin, and azithromycin, respectively (Table 4 and Figure 5).

Interestingly, considerable resistance rates of 21.8% (144/660) and 11.2% (74/660) were demonstrated against vancomycin and linezolid, respectively, which are the most recommended drugs for MRSA (Table 4 and Figure 5), demonstrating the alarming emergence of multidrug-resistant MRSA strains and a potential decline in the efficacy of last-resort antimicrobials. Although none (0%) of the isolates were fully resistant to clindamycin, 93% (614/660) of them showed intermediate resistance, indicating that most strains exhibit reduced susceptibility that may compromise therapeutic effectiveness with potential for emerging full resistance upon selective pressure.

The complete resistance of all isolates (100%) to oxacillin and cefotaxime indicates the widespread distribution of β-lactam resistance, which is typically mediated by the *mecA* gene encoding penicillin-binding protein 2a (PBP2a) with low affinity to β-lactams [41]. Such a finding is consistent with previous reports worldwide that documented high rates of oxacillin resistance among *S. aureus* isolated from raw and RTE meat products, including studies from China [42], Egypt [20], and the USA [31].

Tetracycline and aminoglycosides are commonly used as growth promoters and therapeutic agents in livestock, which may have contributed to the selection and maintenance of resistant strains [43] as reflected in the high resistance rates observed for nitrofurantoin (85.8%), ampicillin (75.6%), gentamicin (60.5%), and tetracycline (51.8%) in the current study. Comparable resistance patterns have been reported, where tetracycline-resistant *S. aureus* recovered from meat products ranged between 40% and 80% [20,25,44].

The high resistance rates observed against β-lactams, aminoglycosides, and tetracyclines suggest that these antimicrobial classes should be prioritized in future antimicrobial stewardship and surveillance programs. Moreover, the detection of vancomycin- and linezolid-resistant isolates emphasizes the need to preserve the efficacy of critically important antimicrobials through prudent-use policies and continuous resistance monitoring.

Moderate resistance rates against ciprofloxacin (38%), trimethoprim/sulfamethoxazole (35.2%), kanamycin (34.8%), and levofloxacin (29.5%) detected herein further confirm the multidrug-resistant (MDR) nature of these isolates. Fluoroquinolone resistance in *S. aureus* has been increasingly reported in foodborne strains [45]. These findings highlight the potential risk of reduced treatment efficacy of commonly used antimicrobials in both human and veterinary medicine.

The remarkable detection of resistance to vancomycin (21.8%) and linezolid (11.2%) represents a particularly concerning finding. The emergence of vancomycin-resistant S. *aureus* (VRSA) has been rarely documented in food sources; on the other hand, reports from Egypt [20], Iran [46], and China [47] have confirmed its occurrence in animal products, suggesting possible horizontal gene transfer from vancomycin-resistant enterococci (VRE).

The low resistance of *S. aureus* isolates to linezolid (11.2%) and rifampin (4.8%) is consistent with a previous study reported for *S. aureus* isolates recovered from RTE foods tested in different countries, including Nigeria [48], Poland [49], and China [50], although none of the isolates recovered from RTE pizza tested in Egypt [20] were resistant to both antibiotics.

Interestingly, none of the isolates (0/660) tested in this study were fully resistant to clindamycin, yet 93% displayed intermediate resistance. Such intermediate resistance patterns highlight the necessity for continuous monitoring of clindamycin susceptibility in *S. aureus* of food origin. Conversely, Wang et al. [51] demonstrated that all (*n* = 23) MRSA isolated from retail food samples, including RTE meat, sold in China were resistant to clindamycin.

The distribution of antimicrobial-resistant *S. aureus* isolates (*n* = 660) among the examined meat samples in the present study (Table 5) showed that isolates from raw meat exhibited higher resistance rates compared to those recovered from RTE meat products. Such elevated resistance in raw meat isolates likely reflects antibiotic selection pressures during primary production, whereas RTE meat isolates mainly represent post-processing contaminants with less direct exposure to antibiotics.

Overall, the data confirm that *S. aureus* isolates of food origin exhibit multidrug resistance (MDR), with β-lactams and nitrofurantoin completely ineffective, while linezolid, rifampin, and vancomycin retained the highest effectiveness, although resistance to vancomycin in a significant proportion of isolates is a serious public health concern. The difference in the resistance patterns found in different studies might be attributed to geographical location, locally approved drugs, and misuse or overuse of antibiotics.

The categorization of *S. aureus* isolates (*n* = 660) into multi-, extensively-, and low drug-resistance and the calculated multiple antibiotic resistance (MAR) index, along with the antimicrobial resistance profiles toward the 15 antimicrobial agents examined, is shown in Table 6. Notably, 8.7% (21/248) and 15.8% (29/184) of *S. aureus* strains isolated from ground buffalo meat and grilled kofta samples, respectively, were categorized as extensively drug-resistant with a MAR index of 0.9, while the great majority of the isolates were categorized as multidrug-resistant (MDR) distributed as 87.9% (218/248), 67.9% (125/184), and 89.5% (204/228) among isolates recovered from ground buffalo meat, grilled kofta, and fried liver samples, respectively with MAR indices ranging between 0.3 and 0.7 based on their resistance profiles against the 15 antimicrobials examined (Table 6).

Alarmingly, the existence of XDR strains in this study showed resistance to 13–14 agents, including last-resort antimicrobials such as vancomycin and linezolid, indicating the emergence of highly resilient clones. At the same time, the majority of isolates (over 80%) were classified as multidrug-resistant (MDR), most frequently resistant to β-lactams, aminoglycosides, tetracyclines, fluoroquinolones, and macrolides.

The higher resistance burden in ground buffalo meat could be attributed to the extensive use of antimicrobials in livestock farming, coupled with increased risk of cross-contamination during processing and grinding. Ready-to-eat products, such as kofta and liver, are likely to be exposed to post-processing contamination, which may explain the persistence of MDR phenotypes, albeit with slightly lower MAR indices compared to raw ground meat. Overall, the detection of both MDR and XDR *S. aureus* in meat products raises serious concerns for food safety and public health, as such isolates may compromise therapeutic efficacy and facilitate the dissemination of resistance genes through the food chain.

The high prevalence of multidrug-resistant (MDR) and extensively drug-resistant (XDR) *S. aureus* isolates observed in the present study may be associated with the long-term and widespread use of antimicrobial agents in livestock production. In Egypt, as in many developing countries, tetracyclines, β-lactams, aminoglycosides, sulfonamides, and fluoroquinolones have historically been among the most frequently used antimicrobial classes for disease treatment, prevention, and, in some situations, growth promotion. Such practices may exert selective pressure that facilitates the emergence and persistence of antimicrobial-resistant bacteria within the food production environment. The high resistance rates detected against oxacillin, ampicillin, gentamicin, tetracycline, and fluoroquinolones in the present study are consistent with this explanation. In recent years, Egyptian authorities have strengthened regulations governing veterinary antimicrobial use and promoted prudent-use strategies to combat antimicrobial resistance. Nevertheless, continued surveillance, improved antimicrobial stewardship, enforcement of withdrawal periods, and implementation of One Health approaches remain essential to limit the dissemination of resistant pathogens through the food chain.

The findings of the present study should also be considered within the framework of the One Health concept, which recognizes the interconnectedness of human, animal, and environmental health in the emergence and dissemination of antimicrobial resistance (AMR) [52]. The high prevalence of MRSA, VRSA, enterotoxigenic *S. aureus*, and multidrug-resistant isolates detected in buffalo-derived food products highlights the potential of the food chain as a vehicle for transmitting antimicrobial-resistant bacteria and resistance determinants from food-producing animals to humans [53]. The occurrence of resistance to critically important antimicrobials, including vancomycin and linezolid, is particularly alarming because these drugs represent essential therapeutic options for severe staphylococcal infections in human medicine [52]. Furthermore, contamination of ready-to-eat foods with resistant *S. aureus* strains underscores the potential public health risks associated with inadequate hygiene practices in food processing and handling. Therefore, effective mitigation of AMR requires a coordinated One Health approach that integrates responsible antimicrobial stewardship in both veterinary and human medicine, enhanced biosecurity measures in animal production systems, improved food safety practices, and continuous surveillance of antimicrobial-resistant pathogens across the animal–food–human interface [54].

A limitation of the present study is that microbiological analyses of ready-to-eat sandwiches were performed on homogenized composite samples rather than on individual components (meat, vegetables, and sauces). Therefore, the precise origin of contamination by *S. aureus*, MRSA, and VRSA could not be determined. Nevertheless, the adopted approach was intended to evaluate the microbiological quality and public health risk of the final product as consumed by the customer. Future investigations employing component-specific analyses would be valuable for identifying critical contamination sources and implementing targeted control measures.

## 4. Conclusions

The present study concluded that the marketed ground buffalo meat and RTE kofta and liver sandwiches in Mansoura, Egypt, exhibited high levels of contamination with multidrug-resistant enterotoxigenic *Staphylococcus aureus*, including MRSA and VRSA strains, posing a serious public health threat to consumers. The higher contamination burden observed in raw ground buffalo meat highlights the need for improved hygienic slaughtering practices, strict sanitation of meat-processing and grinding equipment, maintenance of the cold chain, and prudent use of antimicrobials in food-producing animals. For RTE kofta and liver sandwiches, particular attention should be directed toward adequate cooking, prevention of post-cooking contamination, strict personal hygiene during food preparation and serving, and effective sanitation of food-contact surfaces. From a practical perspective, one low-cost and high-impact intervention that could substantially reduce contamination is the strict separation of raw and cooked foods during handling and serving. The use of dedicated utensils, cutting boards, and food-contact surfaces for cooked products can minimize cross-contamination without imposing significant financial burdens on small-scale vendors. In addition, regular hand hygiene and proper sanitation of food-contact surfaces should be promoted through targeted food safety training programs. The findings of this study provide valuable baseline data for public health authorities and food safety regulators and support the adoption of a One Health approach integrating food safety, antimicrobial stewardship, and continuous monitoring of antimicrobial-resistant foodborne pathogens in animal-derived foods. Future investigations should include sampling of food handlers and food-processing environments to determine their contribution to the transmission and dissemination of *S. aureus*, including MRSA and VRSA, within the ready-to-eat meat production chain.

## Figures and Tables

**Table 2:** *Staphylococcus aureus* counts in raw and cooked buffalo meat products, and the number of samples that exceeded the maximum permissible limit.

Product	No. of Samples Tested	*S. aureus* Count (CFU/g)			Unacceptable Number (%) of Samples that Exceeded the Maximum Limit *
		Minimum	Maximum	Mean ± SE	
Ground buffalo meat	100	3.98 × 10^2^	5.01 × 10^6^	7.94 × 10^4^ ± 5.35 × 10^3 a^	53 (53%)
Kofta sandwiches	100	1.26 × 10^2^	2.0 × 10^5^	2.51 × 10^4^ ± 2.29 × 10^3 b^	38 (38%)
Liver sandwiches	100	0.65 × 10^2^	7.94 × 10^4^	6.31 × 10^3^ ± 4.25 × 10^2 c^	45 (45%)
All products	300	0.65 × 10^2^	5.01 × 10^6^	6.74 × 10^4^ ± 3.15 × 10^3^	154 (51.3%)

* The maximal permissible limit is 10^3^ colony-forming units (CFU)/g for meat products according to Egypt’s National Food Safety Authority (NFSA, 2021). Mean ± SE values with different superscript letters are significantly different (*p* < 0.01).

**Table 3:** Enterotoxin genes (*sea*, *seb*, and *sec*) distribution among *S. aureus* isolates recovered from raw and cooked buffalo meat products (*n* = 600).

Meat Products	No. of Strains Isolated	No. (%) of Enterotoxin-Producing Strains	Type and Incidence of Enterotoxins					
			*sea*	*seb*	*sec*	*sea*, *seb*, and *sec*	*sea* and *sec*	*sea* and *seb*
			No. (%)	No. (%)	No. (%)	No. (%)	No. (%)	No. (%)
Ground buffalo meat	248	117 (47.2)	27 (23.1)	18 (15.4)	12 (10.3)	18 (15.4)	13 (11.1)	29 (24.8)
Kofta sandwiches	184	83 (45.1)	19 (22.9)	15 (18.1)	9 (10.8)	15 (18.1)	12 (14.5)	13 (15.7)
Liver sandwiches	228	82 (36)	9 (11)	21 (25.6)	11 (13.4)	16 (19.5)	6 (7.3)	19 (23.2)
Overall	660	282 (42.7)	50 (16.8)	54 (19.1)	32 (10.8)	49 (16.5)	31 (10.4)	61 (20.5)

**Table 4:** Antimicrobial resistance profiles of *S. aureus* isolates (*n* = 660) recovered from raw ground beef and ready-to-eat sandwich samples.

Antimicrobial	Sensitive	Intermediate	Resistant
**Cefotaxime (CTX)**	0 (0%)	0 (0%)	660 (100%)
**Oxacillin (OX)**	0 (0%)	0 (0%)	660 (100%)
**Nitrofurantoin (NIT)**	0 (0%)	94 (14.2%)	566 (85.8%)
**Ampicillin (AMP)**	161 (24.4%)	0 (0%)	499 (75.6%)
**Gentamicin (GEN)**	153 (23.2%)	108 (16.4%)	399(60.5%)
**Tetracycline (TE)**	283 (42.9%)	35 (5.3%)	342 (51.8%)
**Ciprofloxacin (CIP)**	274 (41.5%)	135 (20.5%)	251 (38%)
**Trimethoprim/Sulfamethoxazole (STX)**	404 (61.2%)	24 (3.6%)	232 (35.2%)
**Kanamycin (K)**	278 (42.1%)	152 (23%)	230 (34.8%)
**Levofloxacin (LEV)**	288 (43.6%)	174 (26.4%)	198 (29.5%)
**Azithromycin (AZM)**	378 (43.6%)	131 (19.8%)	151 (22.9%)
**Vancomycin** **(VA)**	379 (57.4%)	137 (20.8%)	144 (21.8%)
**Linezolid** **(LZ)**	584 (88.5%)	2 (0.3%)	74 (11.2%)
**Rifampin (RIF)**	580 (87.9%)	48 (7.3%)	32 (4.8%)
**Clindamycin (CD)**	46 (7%)	614 (93%)	0 (0%)

Legend: CTX, Cefotaxime; OX, oxacillin; NIT, Nitrofurantoin; AMP, Ampicillin; GEN, Gentamicin; TE, Tetracycline; CIP, Ciprofloxacin; STX, Trimethoprim/Sulfamethoxazole; k, kanamycin; LEV, Levofloxacin; AZM, Azithromycin; LZD, Linezolid; RIF, Rifampin; VA, Vancomycin; CD, Clindamycin.

**Table 5:** Distribution of antimicrobial resistance of *S. aureus* isolates (*n* = 660) recovered from raw ground beef and RTE meat Sandwiches.

*Staphylococcus aureus* Sources	Number of Isolates	Number and (%) of Antibiotic-Resistant Isolates														
		CIP	LEV	GEN	K	AZM	CD	VA	RIF	LZ	TE	STX	OX	NIT	CTX	AMP
**Raw ground buffalo meat**	248	112 (45.2)	92 (37.1)	172 (69.3)	125 (50.4)	68 (27.4)	0 (0)	77 (31)	18 (7.2)	41 (16.5)	150 (60.5)	88 (35.5)	248 (100)	228 (91.9)	248 (100)	205 (82.7)
**Grilled Kofta sandwiches**	184	60 (32.6)	27 (14.7)	110 (59.8)	85 (46.2)	55 (29.9)	0 (0)	50 (27.2)	6 (3.3)	25 (13.6)	148 (80.4)	104 (56.5)	184 (100)	138 (75)	184 (100)	147 (79.9)
**Fried liver sandwiches**	228	79 (34.6)	79 (34.6)	117 (51.3)	20 (8.7)	28 (12.2)	0 (0)	17 (7.5)	8 (3.5)	8 (3.5)	44 (19.3)	40 (17.5)	228 (100)	200 (87.7)	228 (100)	147 (64.5)
**Total isolates**	660	251 (38)	198 (30)	399 (60.5)	230 (34.8)	151 (22.9)	0 (0)	144 (21.8)	32 (4.8)	74 (11.2)	342 (51.8)	232 (35.2)	660 (100)	566 (85.8)	660 (100)	499 (75.6)

CTX, Cefotaxime; OX, oxacillin; NIT, Nitrofurantoin; AMP, Ampicillin; GEN, Gentamicin; TE, Tetracycline; CIP, Ciprofloxacin; STX, Trimethoprim/Sulfamethoxazole; k, kanamycin; LEV, Levofloxacin; AZM, Azithromycin; LZD, Linezolid; RIF, Rifampin; VA, Vancomycin; CD, Clindamycin.

**Table 6:** Prevalence of extensively drug-, multidrug-, and low-drug-resistant *S. aureus* isolates (*n* = 660) based on their antibiotic resistance profile against fifteen antibiotics tested and according to their multiple antibiotic resistance (MAR) index.

Sources	Antimicrobial Resistance Profile	Number	MAR Index	Classification of Strains	
				Type of Resistance	No. and %
Ground buffalo meat (*n* = 248)	CTX, OX, NIT, AMP, GEN, TE, CIP, STX, K, LEV, AZM, LZ, RIF, VA	14	0.933	Extensively drug-resistant	8.47%
	CTX, OX, NIT, AMP, GEN, TE, CIP, STX, K, LEV, AZM, LZ, RIF	7	0.866		
	CTX, OX, NIT, AMP, GEN, TE, CIP, STX, K, LEV, AZM	29	0.733	Multidrug-resistant	87.9%
	CTX, OX, NIT, AMP, GEN, TE, CIP, STX, K, LEV	25	0.667		
	CTX, OX, NIT, AMP, GEN, TE, CIP, STX, K, AZM	5	0.667		
	CTX, OX, NIT, AMP, GEN, TE, STX, K, AZM	7	0.6		
	CTX, OX, NIT, AMP, GEN, TE, STX, AZM	4	0.533		
	CTX, OX, NIT, AMP, GEN, TE, STX, K	3	0.533		
	CTX, OX, NIT, AMP, GEN, TE, AZM	11	0.466		
	CTX, OX, NIT, AMP, TE, CIP	12	0.4		
	CTX, OX, NIT, AMP, TE, LEV	18	0.4		
	CTX, OX, NIT, AMP, K, AZM	7	0.4		
	CTX, OX, NIT, AMP, GEN, CIP	22	0.4		
	CTX, OX, NIT, AMP, TE	11	0.333		
	CTX, OX, NIT, TE, CIP	5	0.333		
	CTX, OX, NIT, AMP, AZM	4	0.333		
	CTX, OX, NIT, AMP	7	0.226		
	CTX, OX, NIT, GEN	18	0.226		
	CTX, OX, NIT, CIP	11	0.226		
	CTX, OX, NIT, LZ	19	0.226		
	CTX, OX, NIT	9	0.20	Low drug-resistant	3.6%
*Average MAR Index for S. aureus = 0.470*					
Grilled kofta (*n* = 184)	CTX, OX, NIT, AMP, GEN, TE, CIP, STX, K, AZM	5	0.875	Extensively drug-resistant	15.8%
	CTX, OX, NIT, AMP, GEN, TE, CIP, STX, K, LEV	24	0.875		
	CTX, OX, NIT, AMP, GEN, TE, STX, K, AZM	11	0.667	Multidrug-resistant	67.9%
	CTX, OX, AMP, GEN, TE, STX, K, AZM, LZ	7	0.667		
	CTX, OX, NIT, AMP, GEN, TE, STX, K, LZ	5	0.6		
	CTX, OX, NIT, AMP, GEN, TE, STX, K	25	0.6		
	CTX, OX, NIT, AMP, GEN, STX, K, AZM	9	0.6		
	CTX, OX, NIT, AMP, GEN, TE, STX, LEV	5	0.533		
	CTX, OX, AMP, GEN, TE, STX, K, AZM	3	0.533		
	CTX, OX, NIT, TE, STX, K, AZM, LZ	3	0.533		
	CTX, OX, NIT, AMP, GEN, K, AZM	4	0.533		
	CTX, OX, NIT, AMP, TE, LZ	10	0.533		
	CTX, OX, NIT, AMP, TE, K	5	0.466		
	CTX, OX, NIT, AMP, TE, RIF	5	0.4		
	CTX, OX, AMP, GEN, CIP	8	0.4		
	CTX, OX, NIT, AMP, TE	7	0.333		
	CTX, OX, GEN, CIP	8	0.266		
	CTX, OX, NIT, TE	10	0.266		
	CTX, OX, NIT, AMP	10	0.266	Low drug-resistant	16.3%
	CTX, OX, AMP, TE	20	0.266		
*Average MAR Index for S. aureus = 0.460*					
Fried liver (*n* = 228)	CTX, OX, AMP, GEN, CIP, STX, LEV, AZM, RIF	8	0.6	Multidrug-resistant	89.5%
	CTX, OX, NIT, AMP, GEN, TE, STX, K	8	0.533		
	CTX, OX, AMP, GEN, TE, STX, K, AZM	5	0.533		
	CTX, OX, NIT, AMP, GEN, CIP, LEV	33	0.466		
	CTX, OX, NIT, AMP, GEN, STX, LEV	8	0.466		
	CTX, OX, NIT, AMP, GEN, CIP, AZM	8	0.466		
	CTX, OX, NIT, AMP, GEN, K, AZM	4	0.466		
	CTX, OX, NIT, AMP, GEN, LEV	17	0.4		
	CTX, OX, NIT, AMP, GEN, STX	8	0.4		
	CTX, OX, NIT, AMP, CIP, LZ	4	0.4		
	CTX, OX, NIT, AMP, TE, K	4	0.4		
	CTX, OX, NIT, AMP, LEV, LZ	5	0.4		
	CTX, OX, NIT, AMP, TE, STX	4	0.4		
	CTX, OX, NIT, AMP, CIP, LEV	4	0.4		
	CTX, OX, NIT, AMP, LEV	6	0.333		
	CTX, OX, NIT, AMP, CIP	4	0.333		
	CTX, OX, NIT, AMP, AZM	4	0.333		
	CTX, OX, NIT, GEN	32	0.266		
	CTX, OX, NIT, TE	21	0.266		
	CTX, OX, GEN, CIP	9	0.266		
	CTX, OX, NIT, CIP	8	0.266		
	CTX, OX, AMP, TE	4	0.266	Low drug-resistant	10.5%
	CTX, OX, NIT	8	0.2		
	CTX, OX, NIT, AMP	12	0.266		
*Average MAR Index for S. aureus = 0.380*					

CTX, Cefotaxime; OX, oxacillin; NIT, Nitrofurantoin; AMP, Ampicillin; GEN, Gentamicin; TE, Tetracycline; CIP, Ciprofloxacin; STX, Trimethoprim/Sulfamethoxazole; k, kanamycin; LEV, Levofloxacin; AZM, Azithromycin; LZD, Linezolid; RIF, Rifampin; VA, Vancomycin; CD, Clindamycin.

## Data Availability

The original contributions presented in the study are included in the article; further inquiries can be directed to the corresponding authors.
